# A uterine‐centric view of reproductive senescence: Genetic insights into late‐life performance in laying hens

**DOI:** 10.1002/imo2.70128

**Published:** 2026-07-30

**Authors:** Junnan Zhang, Mingyue Gao, Ronglang Cai, Xinwei Jiang, Boxuan Zhang, Zhaojie Li, Ning Yang, Congjiao Sun

**Affiliations:** ^1^ State Key Laboratory of Animal Biotech Breeding, Frontier Science Center of Molecular Design Breeding China Agricultural University Beijing China; ^2^ National Engineering Laboratory for Animal Breeding, College of Animal Science and Technology China Agricultural University Beijing China

## Abstract

This study identifies uterus weight as a heritable (*h* ^2^ = 0.45) intermediate phenotype that independently associates with late‐life egg production and eggshell quality in aged laying hens. Genome‐wide association study (GWAS) reveals a genome‐wide significant locus on chromosome 3 and suggestive loci on chromosome 11 for uterus weight, mediation analysis statistically decomposed genetic effects into indirect and direct components, with uterus weight serving as a statistically supported intermediate phenotype, thereby uncovering associations missed by conventional GWAS. Integrative multi‐omics: transcriptome‐wide association study (TWAS), summary‐data‐based mendelian randomization (SMR) and weighted gene co‐expression network analysis (WGCNA) prioritize 11 candidate genes spanning endocrine signaling, lipid metabolism, ion transport, and tissue remodeling. Collectively, these findings establish uterus weight as a biologically central hub connecting genetic variation to reproductive aging, providing potential molecular markers for enhancing laying persistence in extended production cycles.

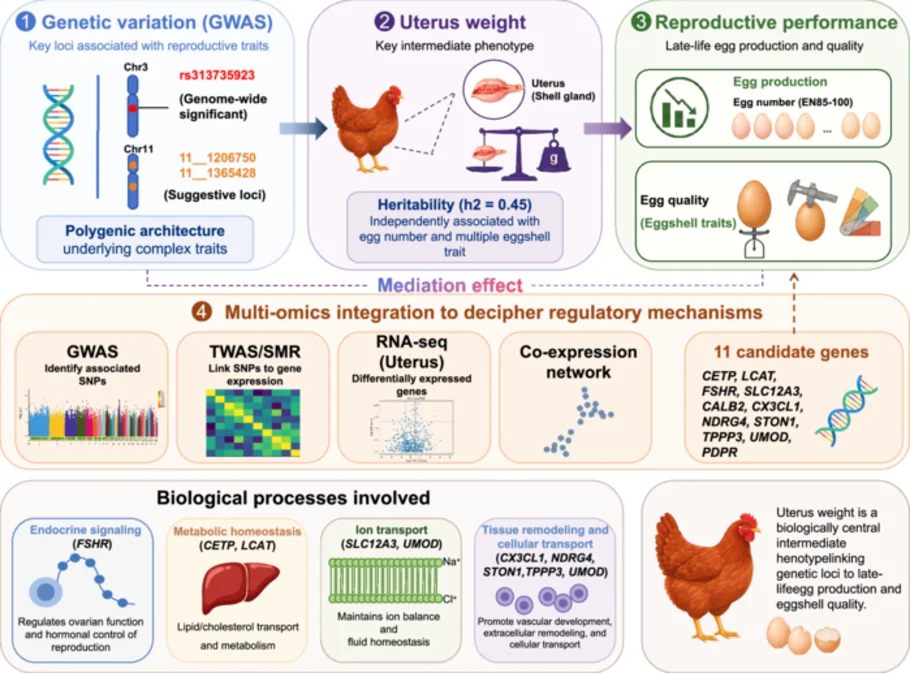

To the Editor,

Egg production and egg quality are economically critical traits in the laying hen industry; however, both deteriorate during the late laying period, which is accompanied by a reduced carcass value of spent hens [1]. With the extension of laying cycles in modern production, aged hens now constitute a significant proportion of commercial flocks [2]. Previous studies have identified genetic parameters, quantitative trait loci, and candidate genes associated with body weight, carcass composition, egg production, and egg quality in poultry [3, 4, 5]. In reality, carcass traits and reproductive traits may share common genetic determinants, particularly during the late laying stage when physiological regulation and resource allocation become more complex [6]. Nevertheless, the genetic relationships among carcass traits, egg production, and egg quality in aged laying hens remain poorly understood.

Complex economic traits are typically regulated by multiple genes and pathways, rendering single‐layer analyses inadequate for uncovering their underlying mechanisms [7, 8]. Genome‐wide association studies (GWAS) have been extensively employed to identify genetic variants associated with economic traits, while transcriptomic analyses offer functional insights at the tissue level [9, 10, 11]. However, conventional GWAS primarily detect direct variant‐phenotype associations and may overlook genetic effects that operate through intermediate biological processes. Intermediate phenotypes may statistically link genetic variation with downstream production traits. Unlike conventional studies that examine carcass or egg traits in isolation, we propose uterus weight as a biologically anchored intermediate phenotype that captures the shared genetic architecture between reproductive aging and eggshell formation. In this study, we investigated the genetic relationships among carcass traits, egg production, and egg quality in aged laying hens using an integrative multi‐omics framework, with a particular emphasis on uterus weight as a potential intermediate phenotype. By integrating GWAS, causal mediation analysis, transcriptome‐wide association study (TWAS), summary‐data‐based Mendelian randomization (SMR), and multi‐tissue expression and network analyses, we aimed to identify genetic variants and candidate genes that regulate uterine development and late‐stage reproductive performance.

## 1 HERITABILITY AND INDEPENDENT ASSOCIATIONS OF UTERUS WEIGHT WITH EGG NUMBER AND EGGSHELL TRAITS

We analyzed a cohort of 254 Rhode Island Red laying hens at 100 weeks of age. Hens exhibited heterogeneous reproductive status, with most individuals still maintaining laying activity but showing varying degrees of reproductive senescence, providing a suitable model for late‐stage reproductive decline. The phenotypic data encompassed 16 traits related to carcass, egg production, and egg quality (Table S1). The uterus weight at 100 weeks (UW100) exhibited substantial variation, with a mean of 15.22 g, a standard deviation of 2.80, and a coefficient of variation of 18.43%. Correlation analysis demonstrated that UW100 was positively correlated with the number of eggs produced from 85 to 100 weeks (EN85‐100, *r* = 0.37, FDR < 0.001) (Figure S1A). The negative correlation between uterus weight and eggshell color lightness (ESCL100) may reflect altered uterine physiological activity that influences pigment deposition during eggshell formation. To account for the overall body size, we employed multivariable linear regression models that included body weight at 100 weeks (BW100) as a covariate. Following the adjustment for BW100, most carcass traits lost their significant associations with egg traits. In contrast, UW100 retained a significant association with EN85‐100 (FDR < 0.001) (Figure S1B). These findings suggest that the impact of uterus weight on reproductive performance in later life is largely independent of body size. Utilizing genome‐wide SNP data, we estimated the heritability of UW100 to be 0.45, indicating a moderate‐to‐high genetic component that supports its consideration as a focal trait in subsequent genetic analyses.

## 2 A GENOME‐WIDE SIGNIFICANT LOCUS ON CHROMOSOME 3 FOR UTERUS WEIGHT

We conducted GWAS utilizing 6.6 million SNPs following rigorous quality control measures. Our analysis revealed no genome‐wide significant association signals for egg production and egg quality traits, suggesting a highly polygenic architecture. However, we identified suggestive peaks for ESCL100 on chromosomes 13 and 21, as well as for eggshell strength at weeks (ESS100) on chromosome 4. In stark contrast, UW100 exhibited a pronounced genome‐wide significant association peak on chromosome 3 (Figure 1A). The surrounding SNPs, which are in moderate‐to‐high linkage disequilibrium, are associated with several genes involved in hormone regulation, including *NRXN1*, *FSHR*, and *LHCGR*. *FSHR* and *LHCGR* are critical components of the hypothalamic‐pituitary‐gonadal axis and are essential for ovarian steroidogenesis and hormonal signaling (Tables S2–S3). Genetic variation in these receptors can affect circulating levels of estrogen and progesterone, which are vital regulators of uterine growth, endometrial remodeling, and tissue maintenance in laying hens. *NRXN1*, primarily recognized for its role in synaptic organization, may indirectly influence these processes through neuroendocrine pathways that affect hormone release or reproductive behavior.

**Figure 1:**
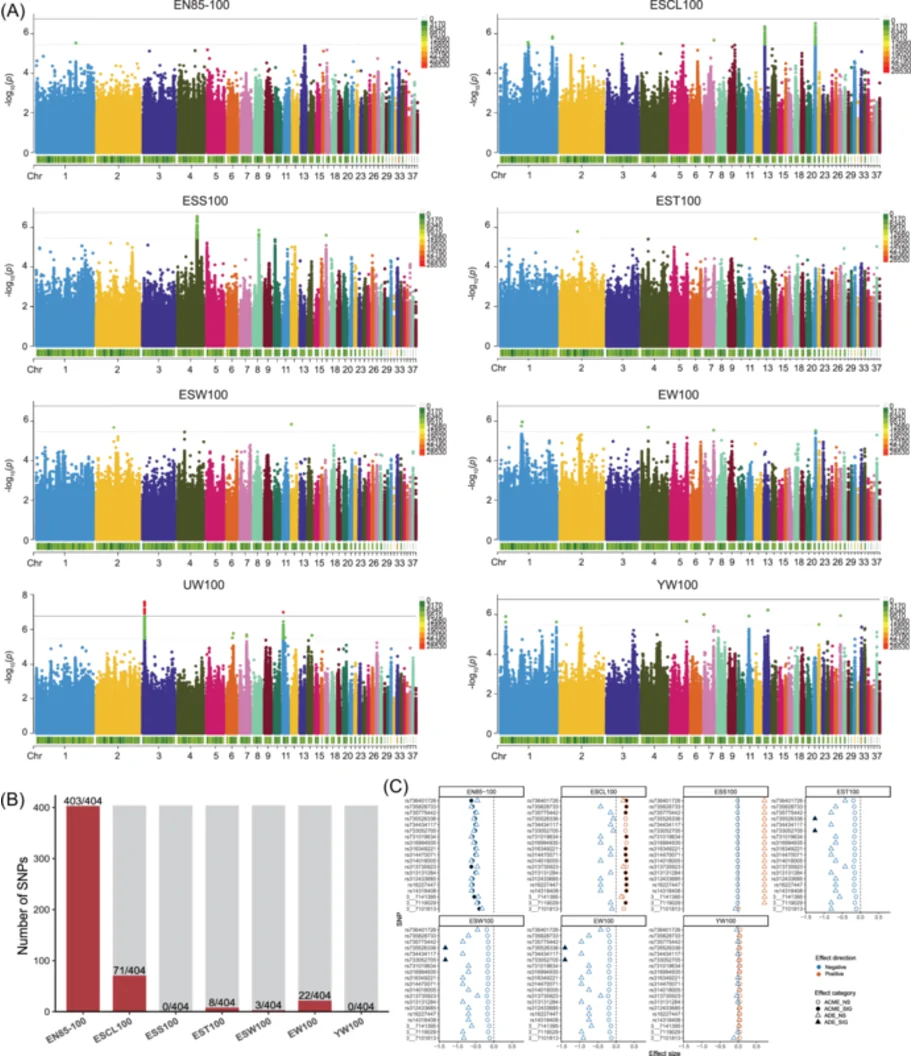
Genome‐wide association patterns and genetic effects statistically decomposed using uterus weight as an intermediate phenotype on egg production and egg quality traits in aged laying hens. (A) Manhattan plots of GWAS for egg production, egg quality, and uterus weight traits measured at 100 weeks of age. The *x*‐axis represents genomic positions across autosomal chromosomes, and the *y*‐axis indicates the negative logarithm of the association *p*‐value (−log_10_(*p*)). Alternating colors denote different chromosomes. The solid horizontal line indicates the genome‐wide significance threshold (0.05/286,983), while the dashed line indicates the suggestive significance threshold (1/286,983). (B) Number of SNPs significantly associated with egg production and egg quality traits that exhibit significant uterus weight‐mediated effects. The numbers above bars indicate the proportion of SNPs with significant mediation effects relative to the total number of analyzed SNPs for each trait. (C) Mediation analysis results for SNPs on chromosome 3 associated with egg production and egg quality traits using UW100 as the mediator. For each SNP, the total genetic effect was decomposed into the average causal mediation effect (ACME; indirect effect mediated through uterus weight) and the average direct effect (ADE; effect independent of uterus weight). The *x*‐axis shows effect sizes, and the *y*‐axis lists SNP identifiers. Point color indicates the direction of the effect (positive or negative), while point shape denotes effect category (ACME or ADE) and statistical significance. 100 means at 100 weeks of age. AFW, abdominal fat weight; BW, body weight; CW, cecum weight; DW, duodenum weight; EN, egg number; ESCL, eggshell color lightness; ESS, eggshell strength; EST, eggshell thickness; ESW, eggshell weight; EW, egg weight; IW, ileum weight; JW, jejunum weight; LW, liver weight; OW, ovary weight; UW, uterus weight; YW, yolk weight.

## 3 STATISTICAL MEDIATION ANALYSIS OF SNP EFFECTS USING UTERUS WEIGHT AS AN INTERMEDIATE PHENOTYPE

Given the phenotypic and genetic associations, we hypothesized that genetic variants may exhibit indirect statistical associations with egg production and egg quality through their influence on uterus weight. To test this hypothesis, we conducted causal mediation analysis using UW100 as the mediator for 404 SNPs that exhibited suggestive associations (*p* < 1/286,983) with any trait. For each SNP, the total genetic effect on an outcome trait was decomposed into an indirect effect mediated by UW100 (average causal mediation effect, ACME) and a direct effect independent of UW100 (average direct effect, ADE). Our analysis revealed that 403 SNPs exhibited significant mediation effects on EN85‐100,71 SNPs on ESCL100, and 8, 3, and 22 SNPs on eggshell thickness at 100 weeks (EST100), eggshell weight at 100 weeks (ESW100), and egg weight at 100 weeks (EW100), respectively (Figure 1B, Table S4). No significant mediation was detected for ESS100 or YW100. Notably, for both EN85‐100 and ESCL100, multiple SNPs showed significant ACME even in the absence of direct GWAS signals, underscoring the power of mediation analysis to uncover genetic relationships that univariate GWAS may overlook. For SNPs located on chromosome 3, the ACME estimates were consistently positive for EN85‐100 and negative for ESCL100, indicating a coherent regulatory direction: genetic variants that increase uterus weight subsequently enhance egg production and lighten eggshell color (Figure 1C). These findings suggest that uterus weight represents a statistically supported intermediate phenotype linking genetic variation with late‐life reproductive performance. The mediation framework used in this study provides a statistical decomposition of genetic effects into indirect and direct components. In this context, uterus weight acts as a biologically motivated intermediate trait linking genetic variation to downstream reproductive phenotypes.

## 4 IDENTIFICATION OF CANDIDATE GENES VIA MULTI‐TISSUE TWAS, SMR, AND EXPRESSION ANALYSES

To identify genes whose genetically regulated expression may be causally associated with UW100, we performed TWAS and SMR across 10 tissues (hypothalamus, pituitary, liver, pancreas, ovary, magnum, uterus, cecum, ileum, duodenum). We identified nine significant gene‐tissue associations corresponding to seven unique genes (Table S5). Among the TWAS/SMR‐identified genes, we further evaluated the direction of effect based on the sign of the TWAS Z‐scores, which indicated whether increased genetically predicted expression was associated with higher or lower uterus weight. Notably, *AARS1* was consistently detected in multiple peripheral tissues (cecum, liver, pancreas), and its top SNP (11_1293953) lies within a suggestive GWAS peak on chromosome 11, with strong local linkage disequilibrium (*R* ^2^ > 0.7). In the hypothalamus, *LCAT* and *CLEC18* were specifically identified, suggesting central regulatory mechanisms. In the uterus, *KCTD19*, *ZBBX*, and an unannotated transcript were detected, highlighting tissue‐specific effects. All SMR associations passed the HEIDI test (*p*_HEIDI > 0.05), indicating shared causal variants.

We further performed differential expression analysis between the top 20 and bottom 20 individuals ranked by UW100. Substantial transcriptional changes were observed in the ovary (481 DEGs), magnum (29 DEGs), and uterus (416 DEGs) (Figure S2A). GO enrichment showed tissue‐specific patterns: extracellular matrix processes were upregulated in the ovary, muscle‑related processes in the magnum, and biomineralization and receptor signaling in the uterus (Figure S2B). UpSet analysis revealed limited overlap of DEGs involved in reproductive system development across different tissues (Figure S2C), indicating that uterus weight‐associated transcriptional regulation is predominantly driven by tissue‐specific expression changes rather than shared regulatory genes. The limited overlap of differentially expressed genes across tissues likely reflects strong tissue‐specific regulatory programs during reproductive aging, highlighting the distinct functional roles of ovarian and uterine tissues. Weighted gene co‐expression network analysis (WGCNA) identified modules significantly correlated with UW100 in the pituitary, liver, duodenum, ovary, and uterus (module eigengene‐trait correlation > 0.4, FDR < 0.05, Figure S2D,E, Figure S3).

Finally, by integrating DESeq2‐based differential expression analyses with WGCNA‐derived gene modules, we identified high‐confidence genes associated with reproductive system development and uterus weight variation (Table S6). UpSet analysis across multiple tissues revealed minimal overlap among these gene sets, suggesting that reproductive system development is predominantly regulated through tissue‐specific transcriptional programs (Figure 2A). Then we integrated GWAS, TWAS/SMR, differential expression, and WGCNA to prioritize 11 candidate functional genes: *CETP*, *LCAT*, *FSHR*, *SLC12A3*, *CALB2*, *CX3CL1*, *NDRG4*, *STON1*, *TPPP3*, *UMOD*, and *PDPR* associated with multiple tissues (Table S7, Figure 2B). These genes were further mapped to major regulatory modules. *CETP* and *LCAT* are involved in cholesterol and lipid metabolism, which are essential for steroid hormone biosynthesis. *FSHR* mediates gonadotropin signaling. *SLC12A3*, *CALB2*, and *PDPR* are linked to ion and energy homeostasis. *CX3CL1*, *NDRG4*, *STON1*, *TPPP3*, and *UMOD* participate in tissue remodeling, angiogenesis, and cellular transport. SNP–gene interaction networks showed that rs31373593 directly connects to *FSHR* and *STON1*, while 11_1206750 connects to *CETP*, *CALB2*, *CX3CL1*, *NDRG4*, *TPPP3*, *UMOD*, and *PDPR*, and 11_1365428 connects to *LCAT* (Figure 2C). These networks demonstrate that multiple independent loci converge on a limited set of biologically relevant genes, coordinating endocrine, metabolic, and structural pathways to influence uterine development and reproductive performance. First, endocrine regulation mediated by gonadotropin signaling pathways, particularly through *FSHR* and *LHCGR*, forms the upstream regulatory axis controlling uterine development and maintenance. Second, steroid hormone‐dependent regulation is closely coupled with calcium transport and mineralization processes required for eggshell formation. Third, age‐associated tissue remodeling processes, including extracellular matrix organization, angiogenesis, and immune modulation, are reflected by genes such as *CX3CL1*, *NDRG4*, *STON1*, and *TPPP3*. Based on functional annotation, the candidate genes associated with these SNPs were further categorized into major regulatory modules (Figure 2D).

**Figure 2:**
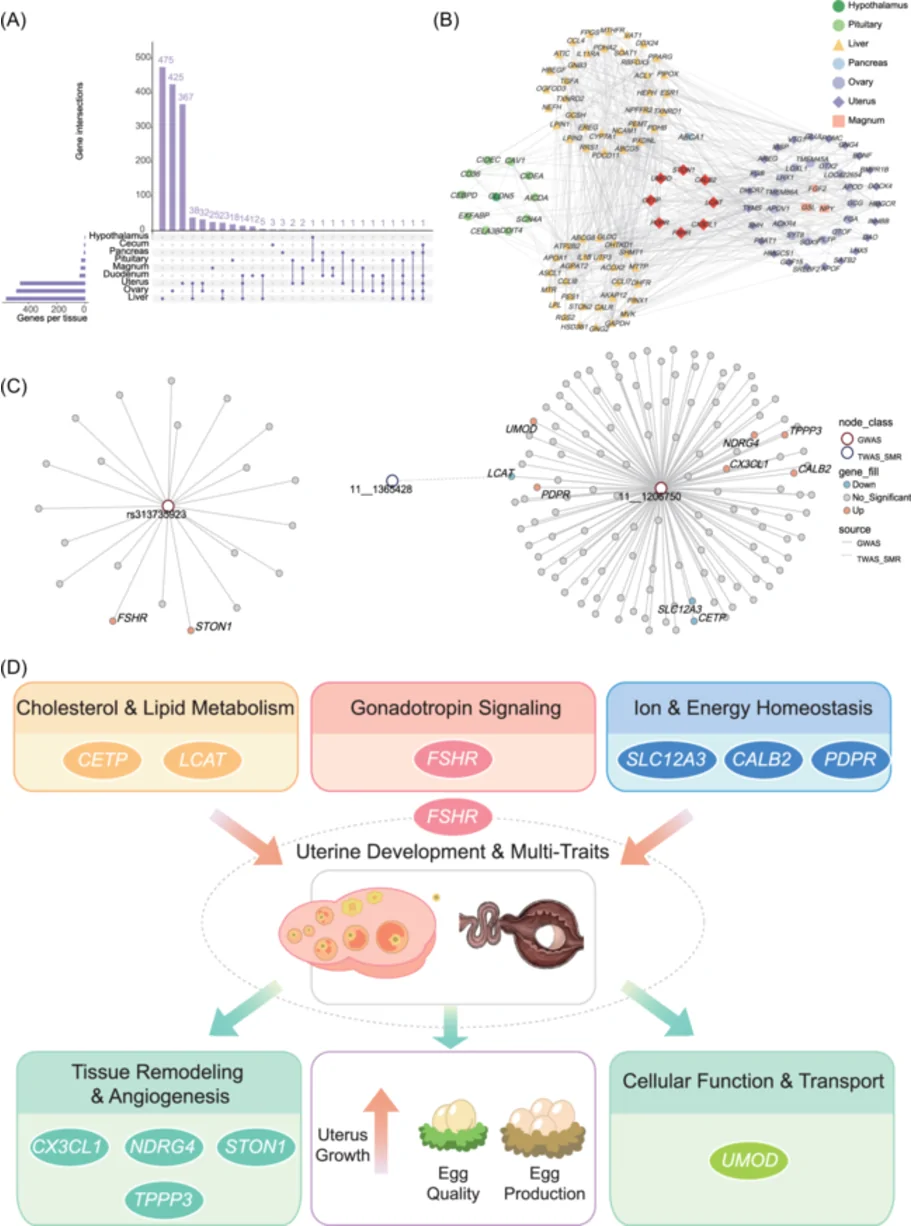
Integrative multi‐omics prioritization of candidate genes associated with uterine development and reproductive performance. (A) UpSet plot showing the overlap of high‐confidence genes identified by integrating DESeq2‐based differential expression analysis and WGCNA across multiple tissues. (B) Cross‐tissue gene interaction network constructed from the integrated differential expression and WGCNA analyses. The red dots represent the core genes. (C) SNP‐gene interaction network integrating GWAS and TWAS/SMR results. (D) Functional classification of the prioritized candidate genes into major biological modules associated with uterine development, including cholesterol and lipid metabolism, gonadotropin signaling, ion and energy homeostasis, tissue remodeling and angiogenesis, and cellular function and transport.

## 5 IMPLICATIONS FOR UNDERSTANDING REPRODUCTIVE AGING AND IMPROVING EXTENDED LAYING CYCLES

Our study provides several key insights. First, uterus weight is identified as a heritable, body weight‐independent intermediate phenotype that encapsulates genetic effects on late‐life egg production and eggshell quality. Second, the selection of uterus weight as a central phenotype was initially motivated by its biological relevance to eggshell formation and its significant associations with multiple reproductive traits. Uterus weight exhibited robust correlations with egg number and eggshell quality traits, and these associations remained significant after adjusting for body weight. Furthermore, uterus weight exhibited a clearer genetic architecture compared to other reproductive traits, supporting its use as a biologically meaningful intermediate phenotype. Third, the 11 prioritized candidate genes underscore the coordinated roles of gonadotropin signaling (*FSHR*), lipid and cholesterol metabolism (*CETP*, *LCAT*), ion and energy balance (*SLC12A3*, *CALB2*, *PDPR*), and tissue remodeling (*CX3CL1*, *NDRG4*, *STON1*, *TPPP3*, *UMOD*) in reproductive aging. And ovarian steroid hormones provide upstream signals that influence uterine calcium transport and tissue remodeling during reproductive aging.

From a practical perspective, although direct selection on uterus weight is impractical in commercial settings, the identified SNPs (e.g., rs313735923, 11_1206750, 11_1365428) and gene expression profiles present potential markers for genomic prediction or indirect selection aimed at enhancing egg production persistence and eggshell quality during extended laying cycles. More broadly, our integrative mediation framework‐merging GWAS, molecular mediation analysis, and multi‐tissue transcriptomics‐serves as a template for unraveling the genetic architecture of complex aging‐related traits in livestock and other species. These analytical approaches are complementary, with GWAS identifying variant‐level signals, TWAS/SMR linking them to gene‐level regulation, and mediation analysis integrating them into phenotype‐level mechanisms.

## 6 CONCLUSION

In conclusion, we demonstrate that uterus weight represents a genetically regulated intermediate phenotype associated with endocrine signaling, metabolic homeostasis, and tissue remodeling to late‐stage reproductive performance. Our findings establish uterus weight as a biologically significant intermediate phenotype and provide prioritized candidate genes and SNPs for enhancing reproductive persistence during extended laying cycles.

## AUTHOR CONTRIBUTIONS

**Junnan Zhang:** Conceptualization; methodology; writing—original draft. **Mingyue Gao, Ronglang Cai,** and **Xinwei Jiang:** Data curation; formal analysis. **Boxuan Zhang** and **Zhaojie Li:** Software; validation. **Ning Yang:** Resources; supervision. **Congjiao Sun:** Conceptualization; supervision; writing—review and editing; funding acquisition.

## CONFLICT OF INTEREST STATEMENT

The authors declare no conflicts of interest.

## AI STATEMENT

The main text was assisted in polishing by DeepSeek V4, and all content had been verified for accuracy by the authors.

## ETHICS STATEMENT

All experiments were approved by the Institutional Animal Care and Use Committee of China Agricultural University (Permit No. AW21214202‐1‐1).

## Supporting information


**Figure S1:** Relationships between carcass traits and egg production and egg quality traits at 100 weeks of age.
**Figure S2:** Integrative multi‐omics analyses identify key genes and regulatory networks associated with reproductive traits.
**Figure S3:** Identification of key modules and functional pathways via WGCNA analysis across multiple tissues.

**Table S1:** Descriptive statistics of 16 traits.
**Table S2:** Linkage disequilibrium analysis of significant SNPs on chromosome 3.
**Table S3:** Genomic annotation of genes located near potential significant SNPs associated with UW100.
**Table S4:** Mediation analysis results for the effects of significant SNPs on egg production and egg quality traits.
**Table S5:** Intersection genes identified by TWAS and SMR analyses across ten tissues.
**Table S6:** The union of genes from the DESeq2 and WGCNA results.
**Table S7:** Candidate functional genes identified by multiple methods.
**Table S8:** The sample sizes for different traits (correlation analysis, heritability assessment, regression analysis, and GWAS).
**Table S9:** The TWAS results of 10 tissues.

## Data Availability

The data that support the findings of this study are available in the supplementary material of this article. Genome and transcriptome data sources were obtained from our previous study, where the detailed experimental setup was described [12, 13, 14, 15]. Sequencing data have been deposited at NCBI under project numbers PRJNA1192671, PRJNA1192672, PRJNA1192674, and PRJNA1263412 (https://www.ncbi.nlm.nih.gov/bioproject/PRJNA1192671, https://www.ncbi.nlm.nih.gov/bioproject/PRJNA1192672, https://www.ncbi.nlm.nih.gov/bioproject/PRJNA1192674, https://www.ncbi.nlm.nih.gov/bioproject/PRJNA1263412). All code could be downloaded from https://github.com/cauzhangjn/Uterus-weight-as-a-mediator-of-egg-production-and-eggshell-quality/tree/main. Supplementary materials (methods, figures, tables, graphical abstract, slides, videos, Chinese translated version, and update materials) may be found in the online DOI or iMeta Science http://www.imeta.science/imetaomics/.
