# Supplementary material for: A uterine‐centric view of reproductive senescence: Genetic insights into late‐life performance in laying hens
Source: IMetaOmics. 2026 Jul 30;3(3):e70128. doi: 10.1002/imo2.70128 (PMC13674417; doi:10.1002/imo2.70128)
Supplement: Supplementary file 1 [file IMO2-3-e70128-s001.docx]

Supporting information to

**A uterine-centric view of reproductive senescence: genetic insights into late-life performance in laying hens**

**Running title**: Uterus weight as a mediator linking to egg production and eggshell quality

Junnan Zhang^1, 2^, Mingyue Gao^1, 2^, Ronglang Cai^1, 2^, Xinwei Jiang^1, 2^, Boxuan Zhang^1, 2^, Zhaojie Li^1, 2^, Ning Yang^1, 2^, Congjiao Sun*^1, 2^

^1^ State Key Laboratory of Animal Biotech Breeding, Frontier Science Center of Molecular Design Breeding, China Agricultural University, Beijing 100193, China

^2^ National Engineering Laboratory for Animal Breeding, College of Animal Science and Technology, China Agricultural University, Beijing 100193, China

Corresponding author:

Congjiao Sun, Email: cjsun@cau.edu.cn.

**METHODS**

**Animals and sample collection**

A total of 254 Rhode Island Red laying hens aged 100 weeks were utilized in this study. All birds were maintained at Beijing Huadu Yukou Poultry Co., Ltd. (Beijing, China) under standardized management conditions, including a controlled photoperiod of 16 h light and 8 h darkness, with free access to feed and water throughout the experimental period. Each hen was individually housed and uniquely identified by wing band and cage number to ensure accurate phenotypic recording. Complete pedigree information was available for all experimental individuals. To minimize physiological variation prior to sampling, all hens were fasted for 24 h before slaughter. On the day of sampling, the birds were humanely euthanized, and tissue collection was performed using a standardized assembly-line protocol. Specifically, each technician was responsible for collecting a single tissue type across all individuals, thereby minimizing technical and batch effects and ensuring anatomical consistency among samples. Blood samples were collected from the wing vein of all 254 hens for genomic DNA extraction. Body weight at 100 weeks of age (BW100) was recorded immediately before slaughter. Following euthanasia, multiple organs and tissues were dissected and weighed, including abdominal fat (AFW100), cecum (CW100), duodenum (DW100), ileum (IW100), jejunum (JW100), ovary (OW100), uterus (UW100), liver (LW100), and gizzard (GW100). For transcriptomic analysis, ten tissues—hypothalamus, pituitary, liver, ovary (site of egg yolk formation), magnum (site of egg albumen secretion), uterus (site of eggshell formation), cecum, ileum, duodenum, and pancreas—were collected from each individual where available. Due to sample quality issues during collection, the final collection yielded a total of 2,474 tissue samples. All samples were immediately snap-collected into RNase-free tubes and stored for subsequent RNA extraction.

**Nucleic acid extraction, quality evaluation, and sequencing**

A. **Genomic DNA Extraction and Sequencing** Genomic DNA was extracted from peripheral blood samples using the TIANamp Genomic DNA Kit (Tiangen, Beijing, China) following the manufacturer’s instructions. The integrity and concentration of the DNA were assessed, and qualified samples were randomly fragmented to achieve an average insert size of approximately 350 bp. The fragmented DNA underwent end-repair processing, 3′ adenylation, and ligation of sequencing adapters. After purification and PCR enrichment, sequencing libraries were generated. Library quality control was performed prior to sequencing. Initial library quantification was conducted using a Qubit 2.0 fluorometer, while the fragment size distribution was examined using an Agilent Bioanalyzer 2100 (Agilent, USA). Accurate library concentrations were further determined by quantitative PCR. Paired-end sequencing (150 bp) was conducted on the Illumina NovaSeq X platform, achieving an average sequencing depth of approximately 10X per individual.
B. **RNA Extraction and Sequencing** Total RNA was isolated from 989 tissue samples (70–100 mg per sample) using the TRIzol reagent (ABI, USA) following standard protocols. RNA concentration and purity were assessed using a NanoDrop 2000 spectrophotometer (Thermo Fisher Scientific, USA). Samples with OD260/280 ratios between 1.8 and 2.1 and OD260/230 values greater than 2.0 were considered suitable for downstream analyses. For transcriptome library construction, mRNA was reverse-transcribed into complementary DNA using the PrimeScript RT reagent kit (Takara, China). The resulting cDNA underwent fragmentation, adapter ligation, and PCR amplification to generate sequencing libraries. Library quantity and quality were evaluated using a Qubit 2.0 fluorometer and an Agilent 2100 Bioanalyzer (Agilent, USA) to confirm concentration and insert size distribution. High-throughput RNA sequencing was subsequently performed on the Illumina NovaSeq X platform using a paired-end 150 bp sequencing strategy.

**Statistical analysis**

A. **Phenotype analysis of different traits** Egg production and quality traits were systematically recorded for all experimental hens. Individual egg number was monitored over the period from 85 to 100 weeks of age (EN85-100). At 100 weeks of age, a series of egg quality traits were evaluated, including eggshell color lightness (ESCL100), eggshell strength (ESS100), eggshell thickness (EST100), eggshell weight (ESW100), egg weight (EW100), and yolk weight (YW100). To ensure measurement reliability, egg quality traits were assessed continuously over three consecutive days, and the mean value across days was used as the final phenotypic record for each hen.
B. **Phenotypic data preprocessing** Phenotypic records for carcass traits, egg production traits, and egg quality traits were obtained from the carcass and laying performance datasets. Individuals with missing values for the corresponding traits were excluded on a trait-by-trait basis. To reduce the influence of non-normal trait distributions on downstream statistical analyses, all phenotypes were evaluated for normality using the Shapiro-Wilk test [1]. When necessary, phenotypes were normalized using an inverse normal transformation (INT), which preserves rank information while improving normality. The distributions of each phenotype before and after INT are presented in figure S4. Transformed phenotypes were used in all subsequent regression and genetic analyses. BW100 was treated as a key covariate and included in regression analysis models to account for overall body size effects.
C. **Multivariable regression analysis** To evaluate the independent contribution of carcass traits to egg production and egg quality traits, multivariable linear regression models were fitted [2]. Specifically, the following model was used:

$$\text{Y}\text{=}\text{β}_{\text{0}}\text{+}\text{β}_{\text{1}}\text{·}\text{carcass}\text{ }\text{traits}\text{+}\text{β}_{\text{2}}\text{·}\text{BW}\text{100+}\sum_{\text{i}\text{=1}}^{\text{k}} \text{r}_{\text{i}}\text{·}\text{PC}_{\text{i}}\text{+}\text{ε}$$

where Y represents the egg production trait or egg quality traits, PCi (i = 1-3) are the first three principal components derived from genome-wide SNP data to correct for population stratification. ε represents the residual error. Regression coefficients, standard errors, and P-values were estimated using ordinary least squares. The statistical significance of carcass traits were used to assess its independent effect after adjusting for body weight. And a FDR < 0.05 was considered significant using the Benjamini-Hochberg procedure.

D. **Mediation analysis of SNP effects on egg production and egg quality** To investigate whether the effects of UW100-associated SNPs on egg production and egg quality traits were mediated through uterine weight, causal mediation analysis was performed using the R package mediation [3, 4]. For each significant SNP-trait pair, two linear regression models were fitted:

Mediator model:

$$\text{Carcass}\text{ }\text{traits}\text{=}\text{α}_{\text{0}}\text{+}\text{α}_{\text{1}}\text{·}\text{SNP}\text{+}\text{α}_{\text{2}}\text{·}\text{BW}\text{100+}\sum_{\text{i}\text{=1}}^{\text{k}} \text{δ}_{\text{i}}\text{·}\text{PC}_{\text{i}}\text{+}\text{ε}_{\text{1}}$$

Outcome model:

$$\text{Y}\text{=}\text{θ}_{\text{0}}\text{+}\text{θ}_{\text{1}}\text{⋅}\text{SNP}\text{+}\text{θ}_{\text{2}}\text{⋅}\text{Carcass}\text{ }\text{traits}\text{+}\text{θ}_{\text{3}}\text{⋅}\text{BW}\text{100+}\sum_{\text{i}\text{=1}}^{\text{k}} \text{η}_{\text{i}}\text{⋅}\text{PC}_{\text{i}}\text{+}\text{ε}_{\text{2}}$$

where SNP denotes the genotype coded additively, carcass traits are the mediators, Y is the egg production or egg quality phenotype, and other terms are defined as above. The average causal mediation effect (ACME), average direct effect (ADE), and the proportion of the total effect mediated by carcass traits were estimated using nonparametric bootstrapping with 1,000 simulations. And a FDR < 0.05 was considered significant using the Benjamini-Hochberg procedure. A significant ACME together with a non-significant ADE was interpreted as evidence that the SNP effect could be statistically decomposed into an indirect component through the intermediate phenotype and a direct component independent of the intermediate phenotype. Given the cross-sectional design, these results should be interpreted as statistical mediation rather than definitive biological or causal mediation.

E. **Whole-Genome Sequencing (WGS) Data Analysis** Sequencing reads were aligned to the chicken reference genome (GRCg7b) via BWA (v0.7.17) [5]. The resulting alignment files were processed with SAMtools (v1.6) to generate sorted BAM files [6]. To minimize potential biases introduced by PCR amplification, duplicate reads were identified and removed using Picard tools (v2.25.2)(https://broadinstitute.github.io/picard/). This step improved the accuracy of downstream variant detection. Single nucleotide polymorphisms (SNPs) were called using the Genome Analysis Toolkit (GATK, v4.2.0). Variant-level quality control was applied using the following filtering criteria: quality by depth (QD) < 2.0, Fisher strand bias (FS) > 60.0, mapping quality (MQ) < 40.0, and variant quality score (QUAL) < 30.0 [7]. All 254 samples passed the quality control procedures and were retained for subsequent analyses. Variant calling was performed in gVCF mode for each individual, followed by joint genotyping to generate a unified VCF dataset. Missing genotypes were imputed using Beagle (v5.4) [8]. Post-imputation filtering was conducted with VCFtools (v0.1.16), retaining SNPs with a minor allele frequency (MAF) greater than 0.01. After quality control, a total of 6,598,746 high-quality SNPs were available for downstream analyses. To reduce linkage disequilibrium and computational redundancy, a subset of 286,983 approximately independent SNPs was selected using PLINK (v1.90) with the parameter --indep-pairwise 50 10 0.2 [9]. Accordingly, SNPs with P values below 0.05/286,983 were considered genome-wide significant, whereas SNPs with P values below 1/286,983 were regarded as suggestively associated with the trait. Genome-wide association analyses were performed using a mixed linear model (MLM) implemented in GCTA (v1.92.2) [10]. For each trait, only individuals with both genotype and phenotype records were included in the analysis; therefore, the effective sample size varied among traits and is summarized in table S8. Principal component analysis (PCA) was performed using the genomic relationship matrix (GRM), and the first five principal components (PC1–PC5) were fitted as fixed-effect covariates to account for population stratification. A sparse genomic relationship matrix, derived from the genome-wide GRM, was included in the mixed linear model to control for genetic relatedness among individuals. All birds were reared under identical management conditions and genotyped in a single batch. Candidate genes located within ±500 kb of SNPs surpassing the small-effect threshold were annotated using BEDTools [11].
F. **RNA-seq Data Processing and Co-expression Analysis** Initial quality control was performed using FastQC (https://www.bioinformatics.babraham.ac.uk/projects/fastqc/). Reads containing more than 10% poly-N were filtered out via fastp (v0.20) to generate clean data [12]. These reads were then aligned to the chicken reference genome (GRCg7b) via HISAT2 (v2.2.1) [13], and gene expression was quantified with StringTie (v2.1.5) [14]. Genes with zero expression in more than 20% of individuals within a given tissue were excluded. The raw count matrix was normalized prior to principal component analysis (PCA). Transcript abundance (TPM) values were log2-transformed to correct for batch effects. Weighted gene co-expression network analysis (WGCNA, v1.71) was conducted using TPM data from the ten tissues [15]. To identify gene modules associated with uterine weight, an extreme phenotype design was adopted. Individuals were ranked according to uterine weight, and the top 20 and bottom 20 individuals were selected for the analysis. These two groups were encoded as binary traits (high = 1 and low = -1), rather than using the original continuous uterine weight values. Pearson correlation coefficients were calculated for all gene pairs, followed by hierarchical clustering via the hclust function to determine the appropriate soft-threshold power (β) for scale-free topology. Gene modules were identified via both one-step and dynamic tree-cutting methods. The module eigengene, defined as the first principal component of each module's expression matrix, was used to assess tissue associations. Modules with a false discovery rate (FDR < 0.05) were considered tissue-associated and retained for further analysis.
G. **eQTL mapping, TWAS and MR analysis** To identify cis-regulatory variants affecting gene expression, gene expression profiles were normalized within each tissue using the trimmed mean of M-values (TMM) method. Genes with low expression (TPM < 0.1 or fewer than six mapped reads in more than 80% of samples) were excluded before association analysis (https://github.com/broadinstitute/gtex-pipeline). Cis-eQTL mapping was performed independently for each tissue using TensorQTL (v1.0.9), incorporating PEER factors, genotype principal components, and additional biological covariates to control for hidden confounding effects. Variants located within ±1 Mb of the transcription start site (TSS) of each gene were tested under the nominal cis-association model. Statistical significance was determined using TensorQTL's permutation framework, in which empirical gene-level P values were first estimated through adaptive permutations and subsequently adjusted across all tested genes within each tissue using the Benjamini-Hochberg procedure. Genes with an FDR < 0.05 were considered significant eGenes. TWAS were performed using S-PrediXcan implemented in the MetaXcan framework. Tissue-specific gene expression prediction models were trained independently for each tissue using matched genotype and RNA-seq data following the PredictDB pipeline (https://github.com/hakyimlab/PredictDB-Tutorial). Prediction models were constructed using cis-eQTLs located within ±1 Mb of each gene., and all successfully trained gene models were retained for downstream association analyses. Consequently, the numbers of tested genes varied among tissues, with 6,336 in cecum, 6,005 in duodenum, 7,034 in hypothalamus, 5,935 in ileum, 6,900 in liver, 3,826 in magnum, 6,330 in ovary, 4,878 in pancreas, 8,225 in pituitary, and 4,657 in uterus. Linkage disequilibrium (LD) covariance matrices required for S-PrediXcan were generated from the corresponding genotype data using PLINK. Statistical significance was evaluated separately within each tissue using the Benjamini-Hochberg procedure, and genes with a FDR < 0.05 were considered significant TWAS associations. No additional filtering based on cross-validation R² was applied, however, detail information was found in Table S9. To further evaluate potential causal relationships between gene expression and complex traits, summary-data-based Mendelian randomization (SMR, v1.3.1) was performed using the cis-eQTL summary statistics generated above. Nominal cis-eQTL results were converted into the BESD format using the options --fastqtl-nominal-format and --make-besd. The same genotype dataset used for eQTL analysis served as the LD reference panel for SMR analyses. Gene-trait associations identified by the SMR test were further examined using the HEIDI test to distinguish pleiotropy from linkage. Associations with significant SMR results and a HEIDI test *P* > 0.05 were considered consistent with a shared causal variant affecting both gene expression and the phenotype. Multiple-testing correction for SMR associations was performed using the Benjamini-Hochberg procedure within each tissue, and an FDR < 0.05 was considered statistically significant.
H. **Functional Enrichment Analysis** Gene Ontology (GO) and KEGG functional enrichment analyses were conducted via clusterProfiler (v4.0) and KOBAS 3.0 [16, 17]. GO term enrichment was performed using the enrichGO function, and KEGG pathway enrichment was performed using the enrichKEGG function. For species-specific annotation, the org.Gg.eg.db and gga databases were used. Enriched terms and pathways were considered statistically significant at an adjust p-value cutoff of 0.05 using the Benjamini-Hochberg procedure.
I. **Visualization** Heatmaps were generated via the pheatmap package (v1.0.12. Bar charts and scatter plots were produced via ggplot2 (v3.4.4), while Venn diagrams were constructed via ggvenn . Manhattan plots were drawn using the CMplot package (v4.5.1) .

**SUPPLEMENTARY FIGURES**

Figure S1. Relationships between carcass traits and egg production and egg quality traits at 100 weeks of age. (A) Correlation analysis between carcass traits and egg production and egg quality traits. The color scale indicates the direction and magnitude of correlations, with red representing positive correlations and blue representing negative correlations. (B) Effects of carcass traits on egg production and egg quality traits after adjustment for BW100. Standardized regression coefficients (β) derived from multiple linear regression models evaluating the independent effects of carcass traits on egg production and egg quality traits after adjustment for BW100. Each point represents the standardized regression coefficient for a given carcass-egg trait pair. The vertical dashed line indicates β = 0. Point colors denote different levels of statistical significance. All traits were measured at 100 weeks of age. ESCL, eggshell color lightness; EST, eggshell thickness; ESS, eggshell strength; ESW, eggshell weight; YW, yolk weight; EW, egg weight; BW, body weight; EN, egg number; AFW, abdominal fat weight; LW, liver weight; OW, ovary weight; UW, uterus weight; DW, duodenum weight; JW, jejunum weight; IW, ileum weight; CW, cecum weight.

Figure S2. Integrative multi-omics analyses identify key genes and regulatory networks associated with reproductive traits. (A) Volcano plots showing DEGs in the ovary, magnum, and uterus. Red and blue dots denote significantly upregulated and downregulated genes, respectively, while gray dots represent non-significant genes. (B) GO enrichment analysis for significant biological processes. Bubble size corresponds to the gene ratio, and color indicates the adjusted p value. (C) UpSet plots illustrating the intersections of DEGs across tissues. (D) Hierarchical clustering dendrogram of genes of uterine tissue. (E) Module-trait relationships. Modules highlighted by the red rectangle meet the selection criteria (module eigengene-trait correlation > 0.4 and FDR < 0.05).

Figure S3. Identification of key modules and functional pathways via WGCNA analysis across multiple tissues. (A) WGCNA results for distinct tissues. From top to bottom: pituitary, liver, duodenum, and ovary. Different colors represent unique gene modules. The module(s) enclosed by a red rectangle met the selection criteria of module eigengene–trait correlation > 0.4 and FDR < 0.05. (B) Bubble plot of GO and KEGG enrichment analyses for genes within the key WGCNA modules. Color intensity represents the level of statistical significance, while bubble size corresponds to the number of genes associated with each term. (C) Network diagram of hub genes clustered by MCODE. Nodes colored in pink denote upregulated genes, and nodes colored in blue represent downregulated genes.

Figure S4. Distribution of phenotypic values before and after inverse normal transformation (INT). Histograms showing the distributions of eight phenotypic traits before (blue) and after (red) INT. Original phenotypic values are shown in the left panel of each trait, and the corresponding INT-transformed values are shown in the right panel.

**REFERENCES**

1. Rafael, Rodrigues de Souza, Marcos Toebe, Anderson Chuquel Mello, Karina Chertok Bittencourt 2023. “Sample size and Shapiro-Wilk test: An analysis for soybean grain yield.” *European Journal of Agronomy* 142: 126666. <https://doi.org/https://doi.org/10.1016/j.eja.2022.126666>

2. Bertha, Hidalgo, Melody Goodman. 2013. “Multivariate or multivariable regression?” *American Journal of Public Health* 103: 39-40. <https://doi.org/10.2105/ajph.2012.300897>

3. Imai, Kosuke, Keele Luke, Yamamoto Teppei. 2010. “Identification, inference and sensitivity analysis for causal mediation effects.” *Statistical Science* 25: 51-71. <https://doi.org/10.1214/10-STS321>

4. Kosuke, Imai, Dustin Tingley, Teppei Yamamoto. 2013. “Experimental designs for identifying causal mechanisms.” *Journal of the Royal Statistical Society: Series A (Statistics in Society)* 176: 5-51. <https://doi.org/https://doi.org/10.1111/j.1467-985X.2012.01032.x>

5. Li, Heng, Richard Durbin. 2009. “Fast and accurate short read alignment with Burrows-Wheeler transform.” *Bioinformatics* 25: 1754-1760. <https://doi.org/10.1093/bioinformatics/btp324>

6. Li, Heng, Bob Handsaker, Alec Wysoker, Tim Fennell, Jue Ruan, Nils Homer, Gabor Marth, Goncalo Abecasis, Richard Durbin; 1000 Genome Project Data Processing Subgroup. 2009. “The Sequence Alignment/Map format and SAMtools.” *Bioinformatics* 25: 2078-2079. <https://doi.org/10.1093/bioinformatics/btp352>

7. Aaron, McKenna, Matthew Hanna, Eric Banks, Andrey Sivachenko, Kristian Cibulskis, Andrew Kernytsky, Kiran Garimella, David Altshuler, Stacey Gabriel, Mark Daly, Mark A DePristo. 2010. “The Genome Analysis Toolkit: a MapReduce framework for analyzing next-generation DNA sequencing data.” *Genome Research* 20: 1297-1303. <https://doi.org/10.1101/gr.107524.110>

8. Brian, L Browning, Ying Zhou, Sharon R Browning. 2018. “A One-Penny Imputed Genome from Next-Generation Reference Panels.” *The American Journal of Human Genetics* 103: 338-348. <https://doi.org/10.1016/j.ajhg.2018.07.015>

9. Shaun, Purcell, Benjamin Neale, Kathe Todd-Brown, Lori Thomas, Manuel A R Ferreira, David Bender, Julian Maller, Pamela Sklar, Paul I W de Bakker, Mark J Daly, Pak C Sham. 2007. “PLINK: a tool set for whole-genome association and population-based linkage analyses.” *The American Journal of Human Genetics* 81: 559-575. <https://doi.org/10.1086/519795>

10. Yang, Jian, S Hong Lee, Michael E Goddard, Peter M Visscher. 2011. “GCTA: a tool for genome-wide complex trait analysis.” *The American Journal of Human Genetics* 88: 76-82. <https://doi.org/10.1016/j.ajhg.2010.11.011>

11. Aaron, R Quinlan, Ira M Hall. 2010. “BEDTools: a flexible suite of utilities for comparing genomic features.” *bioinformatics* 26: 841-842. <https://doi.org/10.1093/bioinformatics/btq033>

12. Chen, Shifu, Yanqing Zhou, Yaru Chen, Jia Gu. 2018. “fastp: an ultra-fast all-in-one FASTQ preprocessor.” B*ioinformatics* 34: i884-i890. <https://doi.org/10.1093/bioinformatics/bty560>

13. Daehwan, Kim, Joseph M Paggi, Chanhee Park, Christopher Bennett, Steven L Salzberg. 2019. “Graph-based genome alignment and genotyping with HISAT2 and HISAT-genotype.” *Nature Biotechnology* 37: 907-915. <https://doi.org/10.1038/s41587-019-0201-4>

14. Mihaela, Pertea, Geo M Pertea, Corina M Antonescu, Tsung-Cheng Chang, Joshua T Mendell, Steven L Salzberg. 2015. “StringTie enables improved reconstruction of a transcriptome from RNA-seq reads.” *Nature Biotechnology* 33: 290-295. <https://doi.org/10.1038/nbt.3122>

15. Peter, Langfelder, Steve Horvath. 2008. “WGCNA: an R package for weighted correlation network analysis.” *BMC Bioinformatics* 9: 559. <https://doi.org/10.1186/1471-2105-9-559>

16. Wu, Tianzhi, Erqiang Hu, Shuangbin Xu, Meijun Chen, Pingfan Guo, Zehan Dai, Tingze Feng, Lang Zhou, Wenli Tang, Li Zhan, Xiaocong Fu, Shanshan Liu, Xiaochen Bo, Guangchuang Yu. 2021. “clusterProfiler 4.0: A universal enrichment tool for interpreting omics data.” *Innovation (Camb)* 2: 100141. <https://doi.org/10.1016/j.xinn.2021.100141>

17. Bu, Dechao, Haitao Luo, Peipei Huo, Zhihao Wang, Shan Zhang, Zihao He, Yang Wu, Lianhe Zhao, Jingjia Liu, Jincheng Guo, Shuangsang Fang, Wanchen Cao, Lan Yi, Yi Zhao, Lei Kong. 2021. “KOBAS-i: intelligent prioritization and exploratory visualization of biological functions for gene enrichment analysis.” *Nucleic Acids Research* 49: W317-w325. <https://doi.org/10.1093/nar/gkab447>
